# Proficiency of European GMO control laboratories to quantify MON89788 soybean in a meat pâté matrix

**DOI:** 10.1016/j.foodcont.2022.109454

**Published:** 2023-03

**Authors:** W. Broothaerts, R. Beaz Hidalgo, G. Buttinger, J. Seghers, M. Maretti, P. Robouch, P. Corbisier

**Affiliations:** a European Commission, Joint Research Centre (JRC), Geel, Belgium; b European Commission, Joint Research Centre (JRC), Ispra, Italy

**Keywords:** Genetically modified organism (GMO), Proficiency test, PCR inhibition, Hot-start PCR, Droplet digital PCR, Meat

## Abstract

GMO control laboratories in the EU routinely monitor the presence and content of genetically modified organisms (GMOs) in food and feed products collected from the EU market. As the vast majority of GMOs comprize genetically modified plants, most control samples have a plant-based origin. For the first time, a pilot proficiency test was organised requiring the analysis of GMOs in a meat matrix. Meat pâté, a product in which soybean is occasionally identified, was spiked with GM soybean event MON89788, homogenised by mixing, aliquoted in sachets and frozen. The assigned value was determined by two independent expert laboratories. Several DNA extraction methods were tested and proved to be insufficient for the removal of PCR inhibitors present in the DNA extracts, resulting in a GM content underestimated by at least 30%. This problem was solved either by using hot-start qPCR chemistry or by applying the same method in a digital PCR format. A total of 52 laboratories participated in the study. They were requested to verify the presence of any GM soybean in the test item and to quantify the GM event(s) identified by their method of choice. All but one laboratory identified the MON89788 soybean event present in the pâté matrix. The majority of the quantitative results reported were below the assigned value, but did not deviate more than 50% from it. This study demonstrated the proficiency of most GMO control laboratories for the analysis of GMOs in a meat-based product. It also shows that method optimisation for GMO analysis in meat products is nevertheless advisable.

## Highlights

• DNA extraction from meat-based materials cannot completely remove PCR inhibitors.
• PCR inhibition is alleviated by use of either hot-start qPCR or droplet digital PCR.
• EU control laboratories are competent for GMO analysis in meat-based products.

## Abbreviations

PT: proficiency test
CRM: certified reference material
CTAB: cetyl trimethyl ammonium bromide
(d)dPCR: (droplet) digital PCR
EURL GMFF: European Union Reference Laboratory for Genetically Modified Food and Feed
H–S qPCR: hot-start qPCR
GM(O): genetically modified (organism)
NRL: national reference laboratory
qPCR: quantitative (or real-time) PCR

## 1 Introduction

Numerous EU official control laboratories routinely test food and feed products for the presence of many chemical substances that could pose a safety risk. In addition, these control laboratories also test for the presence of substances that have been declared safe, such as authorized GMOs, but require testing to verify the correctness of the product labelling and ultimately inform consumers in a transparent way. Indeed, once authorized in the EU, product labelling is required when the GMO is present above a certain threshold (0.9 m/m %). In the EU, nearly all authorized GMOs are GM plants containing inserted recombinant DNA. Legislation enforcement is done at several levels, from feed shipload inspections at harbours to laboratory testing of food products taken from the shelves of supermarkets. Nearly all of the analyzed products have a plant-based origin.

GMO analysis involves homogenisation of the matrix, extraction and purification of DNA and qualitative analysis of the presence of GMOs using PCR screening methods (Bonfini, 2021; Querci et al., 2010; Sabrina et al., 2016). Screening intends to identify both authorized and unauthorized GMOs. When GMO presence is detected in a product, quantitative analysis of the GMO content follows. For each authorized GMO, an event-specific detection method has been validated by the EU Reference Laboratory for GM Food and Feed (EURL GMFF), hosted at the Joint Research Centre of the European Commission (Bonfini et al., 2012). Method validation is done according to minimum performance requirements that are published by the European Network of GMO Laboratories (ENGL). Certified reference materials (CRMs), used for calibration or quality control of the qPCR analysis, are verified by the EURL GMFF and checked for their appropriateness before being made available (https://gmo-crl.jrc.ec.europa.eu/guidance-documents#inline-nav-3). The proper implementation of the analytical methods in the EU national control laboratories is supported and monitored by the EURL GMFF. Since 2010, a proficiency testing (PT) scheme is organised for national reference laboratories (NRLs) and other official control laboratories (Broothaerts et al., 2020). Under this scheme, two test items containing plant GMOs in various food or feed matrices are prepared and distributed twice a year. The participating laboratories report their quantitative results and receive a performance score, which may be shown as a proof of competence to their accreditation body.

The purpose of a PT, organised in line with ISO 17043:2010 (ISO, 2010), is to assess the performance of laboratories for specific tests or measurements and it allows monitoring laboratories' continuous performance. The outcome of a PT may result in the identification of problems in laboratories and initiation of actions for improvement, which, for example, may be related to inadequate test or measurement procedures, effectiveness of staff training and supervision, or calibration of equipment.

In order to enlarge the scope of analysis of the control laboratories towards meat products, a pilot proficiency study was set up in 2021 on the quantification of GM soybean in meat pâté. This study was part of the routine proficiency scheme (Proficiency tests | EURL GMFF (europa.eu)), but the participants were informed that no performance score would be attributed to their results because the majority had no or limited experience with such a matrix. The aim of the pilot proficiency study was to identify and better understand the problems encountered by laboratories when analysing such a matrix and to know to what extent GMO controls in meat products would be reliable.

The meat pâté test item was prepared in line with ISO 17043 requirements. Homogeneity and stability were demonstrated and the value was assigned based on measurements performed by two independent expert laboratories of the JRC. This paper describes the extensive method optimisation required to perform reliable analysis on the DNA recovered from the meat pâté. The results reported by the participating laboratories are evaluated in the light of their competency to perform such analysis as part of their routine control activities.

## 2 Materials and Methods

### 2.1 Materials

Cremepâté (Pâté Crème) was purchased at a local supermarket in Mol, Belgium, and contains pig liver (35%), pig fat, pig meat (18%), rice starch, onion, eggs, milk powder and spices. Bio-Organic (non-GM) Soybeans (https://en.pit-pit.com/) and MON89788 soybean powder (CRM AOCS 0906-B2; www.aocs.org) were ordered online.

### 2.2 Processing

The fresh cream meat pâté was spiked with milled non-GM soybean seeds and GM soybean event MON89788, targeting 12% (m/m) soybean per total mass and a nominal (gravimetrical) MON89788 soybean content of 1.5% (m/m). The GM and non-GM soybean powders were ground to comparable low average particle diameters using a cryo-grinding vibrating mill, then mixed and added to the fresh meat pâté (without the fat layer). The mixture was further homogenised at room temperature using a Stephan UM12 mixer (Gemini B.V., The Netherlands). The mixture was then manually filled into 50 mL glass vials (10 g per vial). Each vial was labelled with an identifier and a unique vial number, placed into an aluminium sachet and stored at −20 °C. The final mixture had a water content of 6.0 ± 0.9 g/100 g (*k* = 2, *n* = 3) and an average particle diameter of 72.2 ± 1.6 μm (*k* = 2, *n* = 3).

### 2.3 DNA extraction

Two independent laboratories, both part of the EURL GMFF (located in Geel, Belgium and Ispra, Italy), analyzed the test item. The frozen pâté was thawed on ice and 200 mg aliquots were taken for DNA extraction. The standard CTAB-based DNA extraction method, used previously for many different matrices, was based on Murray and Thompson (1980) and consists of 1 h lysis at 65 °C in 2% CTAB with RNase A and proteinase K, two chloroform extractions, precipitation with 2% CTAB precipitation buffer, another chloroform extraction following resuspension in high salt solution, isopropanol precipitation and resuspension of the pellet in TE (10 mM Tris-HCl (pH 8.0), 1 mM EDTA).

The CTAB/genomic-tip20 method consists of 1 h lysis at 65 °C in 1% CTAB with RNase A, proteinase K and β-mercaptoethanol, chloroform:octanol (24:1) extraction, precipitation by mixing with 1% CTAB precipitation buffer, resuspension of the pellet in 1.2 M salt, incubation at 50 °C following addition of guanidine-HCl containing Buffer G2 from the Qiagen Genomic-tip 20 kit (Qiagen, Belgium) plus RNase A and proteinase K, and purification of the DNA on a Genomic-tip20 anion-exchange column following the manufacturer's instructions, followed by a final isopropanol precipitation and resuspension in TE_low_ (1 mM Tris-HCl (pH 8.0), 0.01 mM EDTA).

Further modifications for improving the extracted DNA quality consisted of reducing the sample intake to 100 mg, increasing the CTAB content to 2%, prolonging the lysis time to 3 h, and doubling the chloroform:octanol (24:1) extraction.

The amount and the quality of the DNA extracted from the test item were verified by UV spectrometry (Nanodrop™ One, Isogene Life Science, The Netherlands), fluorometry (Quant-iT Picogreen, Invitrogen) and gel electrophoresis. PCR inhibition was tested by qPCR using serial dilutions of the DNA for both the endogenous reference target (four 4-fold dilutions) and the GM event (four 2-fold dilutions) and assessing the slope of the calibration curve (acceptance between −3.1 and −3.6) and comparing the measured and the extrapolated Cq for the undiluted DNA (acceptance ΔCq < 0.5).

### 2.4 PCR

The quantitative detection method for soybean event MON89788, validated by the EURL GMFF (https://gmo-crl.jrc.ec.europa.eu/gmomethods), was used (QT-EVE-GM-006), calibrated with DNA extracted from the official CRM AOCS-0906-B2. The GM percentage is expressed in relation to the lectin endogenous soybean reference target (QT-TAX-GM-002 or -009).

Quantitative PCR (qPCR) was performed in a QuantStudio 7 (QS7) instrument (ThermoFisher Scientific, Belgium). The PCR reaction contained 2X TaqMan Universal PCR Master mix (with UNG; Thermo Fisher Scientific, USA), primers and probe for lectin at a final concentration of 200 nM and for MON89788 at 150 nM and 50 nM for primers and probe, respectively. Hot-start qPCR was performed with JumpStart Taq ReadyMix (Merck KGaA, Germany) in the presence of 1.5 or 3.5 mM MgCl_2_.

Standard qPCR thermal conditions in the QS7 instrument were used, consisting of 2 min at 50 °C, 10 min at 95 °C, followed by 45 cycles of 15 s at 95 °C and 1 min at 60 °C. The ramping speed was 1.6 °C/s.

### 2.5 Droplet digital PCR

ddPCR was performed on the QX200™ Droplet Digital™ PCR system (Bio-Rad, Belgium) using an automatic droplet generator (Bio-Rad, Belgium). The DNA extracts were diluted to 40 or 60 ng/μL. The PCR mix contained ddPCR Supermix for probes (Bio-Rad) and the same final concentration of primers and probe as used for qPCR. PCR thermal conditions were the same as for qPCR, with an unlimited step at 4 °C at the end. The ramping speed was 2.5 °C/s.

After PCR amplification, the droplets were analyzed in a QX200™ droplet reader (Bio-Rad, Belgium), and the absolute quantification of PCR targets was analyzed using QuantaSoft™ software version 1.7.4.0917 with a threshold placed just above the negative droplet cloud. The copy number concentration (cp/μL) was calculated and converted in mass fraction (m/m %) using the conversion factor (0.981 ± 0.021) published on the EURL GMFF webpage (CF-CRM-values.pdf (europa.eu); Corbisier & Emons, 2019; Corbisier et al., 2022).

### 2.6 Characterisation

The homogeneity of the test item was evaluated on 7 vials and 5 replicates (each 200 mg) per vial, according to ISO 13588:2015, Annex B.3 (ISO, 2015). The test item was stored and dispatched frozen, therefore the uncertainty related to its stability during transport was considered negligible and set to zero. Repeated thawing and freezing (tested up to 5 times) did not change the measured GM content. The test item was found stable until the end of the study. The value assigned to the test item was determined as the mean of three datasets comprising 15 (*N* = 5, *n* = 3, i.e. 5 bottles, 3 sample replicates from each bottle) or 35 (*N* = 7, *n* = 5) test results obtained using either hot-start qPCR (2 datasets) or ddPCR (1 dataset). The measurement uncertainty was estimated according to ISO 13588:2015, section 7.2 (ISO, 2015).

### 2.7 Pilot proficiency test

The EU NRLs were invited to participate in this study by registration through an in-house developed online tool for inter-laboratory comparisons (MILC). They were allowed to invite other official control laboratories within their national network. The test items were dispatched on dry ice and had to be stored frozen upon arrival in the laboratories. Detailed instructions were provided and the laboratories were free to use either their routine methods or perform additional method optimisation prior to analysis. The results had to be reported within 6 weeks using the online MILC reporting tool and further details had to be provided in a questionnaire using EU Survey. The reported results were evaluated using estimates of deviation (*D%*, difference between the reported result $x_{i}$ and the assigned value $x_{pt}$ in line with ISO 13588:2015, section 9.3 (ISO, 2015), and they were communicated to the participants in a workshop and through a report.

$$D\%=100*\frac{\left(x_{i}-x_{pt}\right)}{x_{pt}}\;,expressed\;in\;\%$$

## 3 Results and discussion

### 3.1 Characterisation of the test item

The following factors complicated the analytical measurements for the characterisation of the pâté test item:

(i) Genomic DNA extracted from meat samples is known to contain compounds that may inhibit PCR, resulting from the manufacturing processes and/or incompletely removed from the matrix during extraction (Piskata et al., 2019; Schrader et al., 2012; Sidstedt et al., 2020). These compounds may interfere with the PCR by reducing the activity of the Taq DNA polymerase;
(ii) The DNA extracted from the pork meat in the pâté is present in large excess in comparison to the DNA from the spiked soybean, both because of the low soybean fraction in the test item and due to the larger DNA extractability from animal tissues compared to plant tissues (own experience); and
(iii) PCR interference may be due to sequences in the pig genome that are at least partially identical to the primers used in the MON89788 detection method, e.g. BLASTn analysis shows that 17 out of 20 bp of the forward primer and 17 out of 19 bp of the reverse primer are found in pig DNA sequences and, using Primer-BLAST, potential amplicons of 314 bp and more are identified (data not shown).

**Fig. 1:**
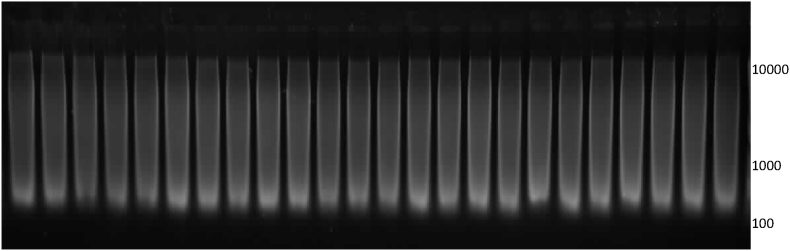
Agarose gel electrophoresis of 24 samples of genomic DNA extracted from the pâté material. The approximate DNA marker sizes (in bp) are indicated on the right.

**Fig. 2:**
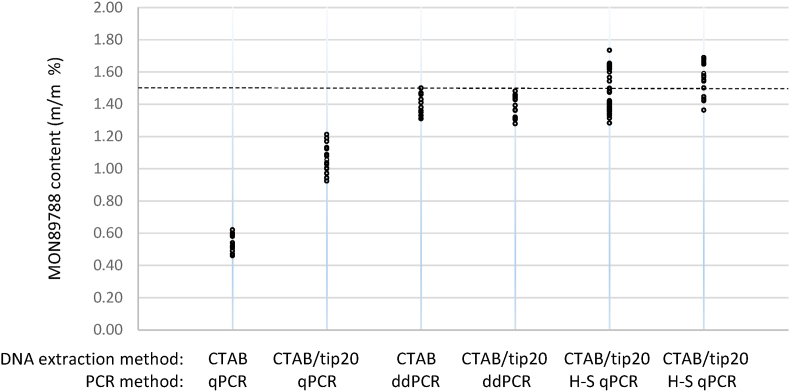
Combined effect of DNA extraction method and PCR method for MON89788 detection in meat pâté. Each method was tested on 15 DNA extracts (35 for column 5). Columns 1 and 3, and columns 2, 4 and 6, show the results obtained in the same laboratory on identical DNA extracts analyzed by different PCR techniques. Dashed line: expected GM %.

The DNA extracted from the test item was of a high concentration (100–300 ng/μL based on Picogreen measurements) and was visible as a long smear on gel electrophoresis, indicating partial degradation (Fig. 1). Initial attempts to extract genomic DNA of PCR-grade quality from the meat pâté test item failed as shown by the PCR inhibition tests. The DNA extraction procedure was therefore modified to reduce the amount of PCR inhibiting or interfering compounds remaining in the DNA extracts. A routine CTAB method involving several chloroform extractions did not yield high quality DNA. When used in qPCR, a GM content far below the expected GM % was measured (Table 1, Fig. 2). DNA extracted by a CTAB method followed by an additional purification on a Genomic-tip20 column produced DNA of acceptable quality based on spectrophotometric measurements (A_260 nm_/A_280 nm_ and A_260 nm_/A_230 nm_ ratios above 1.9 and 2.3, respectively), inhibition tests for the soybean lectin reference gene (Hougs et al., 2017) and qPCR results. Reducing the sample intake from 200 to 100 mg and extending the lysis time from 1 to 3 h further improved the results. However, using a serial dilution series, inhibition tests for the MON89788 target failed for around half of the DNA extracts. This could be due to the low amount of the GM target in the test item, resulting in high and therefore less stable Cq values in the more diluted DNA samples. Further modifications of the extraction procedure did not improve the quality of the DNA. For instance, Poms et al. (2001) applied an *n*-hexane pre-treatment and several modifications to the CTAB extraction method to purify DNA from soybean spiked into different milk fractions, another animal matrix known for its abundance of potential PCR inhibitors. Here, an *n*-hexane treatment prior to the CTAB/genomic-tip20 method did not change the qPCR results, nor did an additional NucleoSpin Food kit (Macherey Nagel, Germany) purification following the CTAB/genomic-tip20 extraction. DNA extraction from the material using commercial NucleoSpin Food or Biotecon (Biotecon Diagnostics, Germany) kits also yielded DNA of comparable quality and GM content as obtained with the optimised CTAB/genomic-tip20 method. The modified CTAB/genomic-tip20 extraction method with a reduced sample intake of 100 mg, using a 2% CTAB buffer and 3h lysis incubation step was chosen for further analyzes.

**Table 1:** Effect of PCR methodology on the measured MON89788 soybean content (average ± *U*, *k* = 2, in m/m %) in the pâté test item (expected GM % = 1.5 m/m %).

Laboratory	DNA extraction method	No. of bottles	No. of replicates	Measured GM content by		
				qPCR	H–S qPCR	ddPCR
Lab 1	CTAB	5	3	0.54 ± 0.05		1.40 ± 0.04
Lab 2	CTAB/tip20	5	3	1.06 ± 0.09	1.56 ± 0.06	1.38 ± 0.07
Lab 2	CTAB/tip20	7	5		1.47 ± 0.04

Because of the partial similarities of the MON89788 primers to sequences found in pig DNA, primer depletion may be another potential source of PCR interference. Doubling the primer and probe concentration, however, did not affect the Cq values measured (data not shown).

In general, PCR inhibition can be overcome by diluting the matrix several fold. However, this approach also dilutes the DNA template, which compromises sensitivity. To alleviate potential PCR inhibition problems, alternative approaches known to be less sensitive to PCR inhibition/interference were tested. First, DNA extracted by the CTAB method was further purified with an additional passage on a purification column (CTAB/Tip 20). This extra purification increased the average GM content measured by qPCR by a factor of 2 (Fig. 2, Table 1) but was not needed for ddPCR. Secondly, the master mix was changed and a hot-start (H–S) master mix was used instead of the classical master mix. Hot-start PCR uses an antibody-inactivated hot-start Taq DNA polymerase designed to minimize non-specific amplification while increasing target yield (Paul et al., 2010). The average GM content measured on 15 DNA extracts using standard qPCR increased by more than 45% when using hot-start qPCR. Similarly, using ddPCR, known to be less affected by PCR inhibitors (Sedlak et al., 2014; note that ddPCR also employs a hot-start polymerase), the average GM content measured in these DNA extracts increased by 30% compared to standard qPCR. Similar GM content was measured by ddPCR on CTAB-extracted DNA obtained without or with a tip20 purification step and more than 2.5 times higher GM content was measured compared to standard qPCR. It was concluded that both approaches, hot-start qPCR and ddPCR, resulted in MON89788 results close to the nominal and likely true GM concentration. In contrast, when using the standard qPCR detection method for MON89788 a negative bias of 30–40% was observed for this particular test item.

The homogeneity of MON89788 soybean in the test item was confirmed using hot-start qPCR. The material was dispatched frozen to ensure stability during transport. The long-term stability over the period of the study was also found negligible. The assigned value for this test item was determined using hot-start qPCR and ddPCR results (Table 2). The associated standard measurement uncertainty of the assigned value (*u(*$x_{pt}$)) was calculated following the law of uncertainty propagation, combining the standard measurement uncertainty of the characterisation (*u*_*char*_) with the standard uncertainty contributions from homogeneity (*u*_*hom*_) and stability (*u*_*stab*_, set to zero), in compliance with ISO 13528:2015, section 7.2 (ISO, 2015).

**Table 2:** Assigned value (*x*_*pt*_) and measurement uncertainty (number of independent measurements in parentheses).

Test item	GM event	PCR method	Average per dataset ± *U* (*k* = 2)	$x_{pt}$	*u*_*char*_	*u*_*hom*_	*u(x*_*pt*_*)*
Meat pâté	MON89788	Hot-start qPCR (35)	1.47 ± 0.04	**1.47**	0.05	0.03	**0.06**
		Hot-start qPCR (15)	1.56 ± 0.06				
		ddPCR (15)	1.38 ± 0.04				

### 3.2 Evaluation of the results

A total of 52 laboratories from across Europe participated to the study. All but one laboratory identified the correct GM event in the test item by application of a series of screening methods and/or GM event-specific methods. The laboratory that failed to detect this GM event mentioned that the isolated DNA was not suitable for further PCR analysis.

**Fig. 3:**
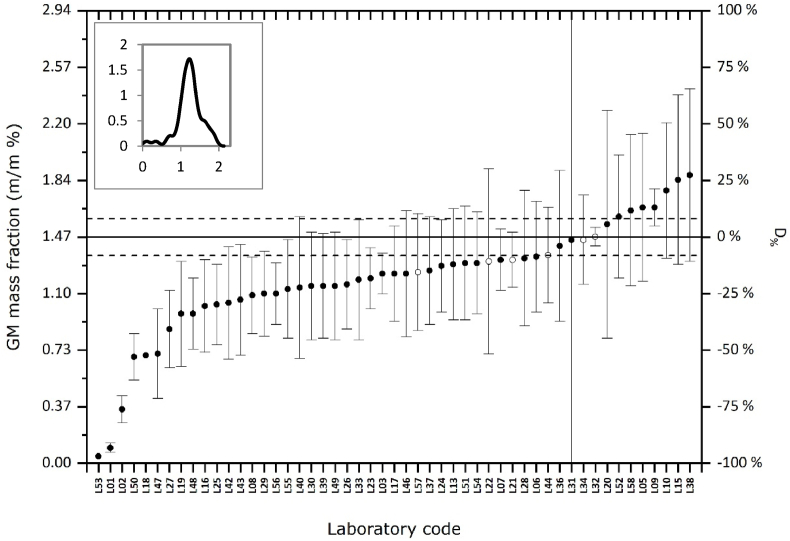
Reported results expressed in GM mass fraction (left y-axis) and their evaluation in *D%* (right y-axis). Horizontal line at 1.47 m/m % corresponds to the assigned value with its expanded measurement uncertainty (dashed lines). Open circles refer to results obtained by dPCR. The kernel density distribution is shown in the high left corner.

The quantitative results reported for MON89788 were evaluated by calculating the deviation from the assigned value (*D%*, see Materials and Methods). In general, all results but two (*i.e.* 96 %) deviated less than 53% from the assigned value, which seems acceptable for this difficult matrix. Over 80% of the results were lower than the assigned value and only 8 reported values (17%) were higher (Fig. 3). Six laboratories used dPCR for quantitation. The *D%* values calculated from the reported dPCR results were all relatively small (below 15%), thus confirming our observations that dPCR is less sensitive to PCR inhibition.

Details on the analysis performed by the laboratories were communicated in a questionnaire accompanying the study. For the analysis on meat pâté, half of the respondents declared to have some experience with DNA extraction from such matrix, but not necessarily in the frame of GMO control. The other participants mentioned to have no experience with such a matrix. A large majority of participants used their routine method for DNA extraction, sometimes with minor modifications, e.g. hexane pre-treatment to remove the fat, extended lysis time (to 3 h) and further clean-up of the DNA with a commercial kit. Most laboratories started from 200 mg sample intake, while some used 500 mg or more. The majority of laboratories (20) applied the NucleoSpin Food kit for DNA extraction, whereas 19 laboratories applied a method based on CTAB. Absence of inhibition in the DNA extracts was evaluated by 25 laboratories using a dilution series and measuring the lectin reference gene, whereas 33 laboratories tested two or more DNA dilutions in the PCR experiments. Nearly all laboratories (47) reported that the DNA extracted from the meat pâté was considered suitable for quantitative analysis. A few laboratories found the DNA suitable only when extracted with one of their applied extraction methods, or when sufficiently diluted (to mitigate the effect of inhibitors).

The results of this pilot proficiency study demonstrated the competence of the participating laboratories to test and quantify GMOs in a challenging meat-based product. When provisionally calculating *z* scores (*z* = $(x_{i}$– $x_{pt}$/*σ*_*pt*_), applying a standard deviation of proficiency testing (*σ*_*pt*_) of 25% of the assigned value, 47 of the 51 laboratories would have received an acceptable performance score. At the same time, this study showed that most laboratories have somewhat underestimated the GM content in this material as the result of using a non-adapted DNA extraction method and/or not using hot-start qPCR or ddPCR to alleviate remaining PCR inhibitors in the extracted DNA. In routine analysis, the presence of inhibiting or interfering PCR compounds in a DNA extract is not always easily detected, e.g. also Cankar et al. (2006) reported a lower PCR efficiency for meat products containing soybean. In particular for new and special matrices like meat-based products it is advised to check for potential inhibition with appropriate procedures. In case of doubt, approaches similar to the ones described here could be applied to assess the validity of the measurement system used.

## CRediT authorship contribution statement

**W. Broothaerts:** Conceptualization, Formal analysis, Writing – original draft, Supervision, Project administration. **R. Beaz Hidalgo:** Methodology, Investigation, Writing – review & editing. **G. Buttinger:** Methodology, Investigation, Writing – review & editing. **J. Seghers:** Resources. **M. Maretti:** Investigation. **P. Robouch:** Conceptualization, Validation, Formal analysis, Writing – review & editing, Visualization. **P. Corbisier:** Validation, Supervision, Writing – review & editing.

## Declaration of competing interest

None.

## Data Availability

The data that has been used is confidential.
